# Normalization of electroretinogram and symptom resolution of melanoma-associated retinopathy with negative autoantibodies after treatment with programmed death-1 (PD-1) inhibitors for metastatic melanoma

**DOI:** 10.1007/s00262-021-02875-x

**Published:** 2021-02-05

**Authors:** Karam Khaddour, Sangeeta Khanna, Michael Ansstas, Ishaan Jakhar, Sonika Dahiya, Laurin Council, George Ansstas

**Affiliations:** 1 Division of Medical Oncology, Washington University in Saint Louis, 660 South Euclid Avenue, Saint Louis, MO 63110 USA; 2 Department of Ophthalmology and Neurology, Saint Louis University, St Louis, MO USA; 3 Allergy and Immunology, Barnes Jewish Christian Health Care, Saint Louis, MO USA; 4 UMKC School of Medicine, Kansas City, MO USA; 5 Division of Neuropathology, Department of Pathology and Immunology, Washington University in Saint Louis, Saint Louis, USA; 6 Division of Dermatology, Washington University in Saint Louis, Saint Louis, USA; 7 Alvin J. Siteman Cancer Center, Saint Louis, MO USA

**Keywords:** Melanoma-associated retinopathy, Programmed death-1 inhibitors, Metastatic melanoma, Pembrolizumab, Paraneoplastic, Autoantibodies

## Abstract

Melanoma-associated retinopathy (MAR) is a paraneoplastic syndrome that involves the production of autoantibodies which can cross-react with retinal epitopes leading to visual symptoms. Autoantibodies can target intracellular proteins, and only a few are directed against membrane proteins. This discrepancy in autoantibody–protein target can translate into different immune responses (T-cell mediated vs B-cell mediated). Historically, treatment of MAR has focused on surgical reduction or immunosuppressive medication, mainly glucocorticoids. However, tumor resection is not relevant in metastatic melanoma in which MAR is mostly encountered. Moreover, the use of glucocorticoids can reduce the efficacy of immunotherapy. We report the first case to our knowledge with subjective resolution of visual symptoms and objective evidence of normalization of electroretinogram of MAR with undetectable autoantibodies after administration of programmed death-1 (PD-1) inhibitor (pembrolizumab) without the use of surgical reduction or systemic immunosuppression. This case highlights the potential improvement and resolution of negative autoantibody MAR with the use of PD-1 inhibitors and emphasizes the importance of multidisciplinary approach and team discussion to avoid interventions that can decrease immunotherapy-mediated anti-tumor effect.

## Introduction

Melanoma-associated retinopathy (MAR) is a rare paraneoplastic syndrome characterized by photopsia and decreased night vision (nyctalopia) initially with normal fundus exam followed by painless progressive vision loss with viewable retinal changes [1]. This paraneoplastic syndrome is caused by the production of autoantibodies against tumor cells that can cross-react with bipolar cells (especially the ON-bipolar cells of rod photoreceptors), which causes degeneration of the ON-bipolar cells by recognizing intracellular retinal proteins as antigens and targeting them [1].

The treatment of malignant melanoma has been revolutionized since the introduction of immune checkpoint inhibitors (ICI) which have replaced chemotherapy and are now considered the standard of care for advanced and metastatic cutaneous melanoma [2, 3]. The mechanism by which ICI exert their effect against melanoma cancer cells is mediated by the blockade of programmed death-1/programmed death-ligand 1 (PD-1/PD-L1) receptors or cytotoxic-associated lymphocyte antigen-4 (CTLA-1) receptors. These receptors (PD-1/PD-L1/CTLA4) are expressed in the tumor microenvironment and offer an immune escape mechanism for the malignant cells leading to suppression of the immune system. By targeting immune checkpoint receptors, ICI can restore the adoptive and adaptive immunosurveillance which lead to elimination of malignant cells [4].

Treatment of MAR has been based on either cytoreduction of the primary tumor or the immunomodulation and/or immunosuppression with medications such as glucocorticoids and intravenous immunoglobulins (IVIG). Of importance, there is a concern with a growing evidence that the use of immunosuppression such as glucocorticoids might counteract the anti-tumor effect of ICI and lead to low response rates to immunotherapy especially if it is started prior to the initiation of ICI [5, 6]. Moreover, there is a concern of exacerbating underlying paraneoplastic symptoms with the use of ICI given that syndromes associated with autoantibodies to intracellular antigens are T-cell mediated through further activation of T-cell compartment [7]. Conversely, paraneoplastic syndromes associated with autoantibodies to cell surface or synaptic proteins are B-cell mediated suggesting that the use of ICI could potentially improve underlying paraneoplastic syndromes through reduction of tumor volume, thus reducing B-cell compartment reactivation [7].

We report our multidisciplinary approach among medical oncologists and ophthalmologists that led to avoiding glucocorticoid administration prior to initiation of cancer treatment with ICI in a patient who was found to have MAR that led to the diagnosis of metastatic melanoma. Interestingly, we observed that patient’s visual symptoms secondary to paraneoplastic syndrome of MAR improved after starting pembrolizumab. This was accompanied by a resolution of electroretinogram (ERG) findings (a hallmark of MAR). This observation provides an insight into the heterogeneous immunological etiology of MAR and warrants further investigation on the safety and efficacy of ICI in MAR subtypes (humoral B-cell-mediated response vs T-cell-mediated response).

## Case presentation

A 74-year-old Caucasian male presented to the ophthalmology clinic with complaints of vision changes. His medical history was significant for paraplegia due to spinal trauma, melanoma of the left upper arm four years prior (Stage I: T1aN0M0) with Breslow depth of 0.77 mm without significant mitotic activity or associated ulceration. An uncomplicated wide local excision was performed at that time without a sentinel lymph node biopsy. He had regular and reassuring dermatological examinations for three years after surgical excision until one year prior to presentation when he was lost to follow-up. His other medical history included atrial fibrillation and hypothyroidism, and there was no history of previous autoimmune disease.

The patient described his vision changes as shimmering lights in the entire field of vision for the past 5 weeks prior to his presentation. The shimmering lights began in his right eye but eventually became bilateral and was accompanied by a decrease in night vision (nyctalopia). The ophthalmologic history was significant for a cataract surgery in the left eye. On examination, he had cataract in the right eye with decreased bilateral visual acuity. His color vision was normal, and no field loss was detected on clinical testing by confrontation. He could not perform reliable automated perimetry since he was wheelchair bound and could not be positioned accurately during the test. The fundus examination revealed a normal optic disc without retinal pigmentary change or arterial attenuation. Optical coherence tomography (OCT) of the maculae was normal.

**Fig. 1:**
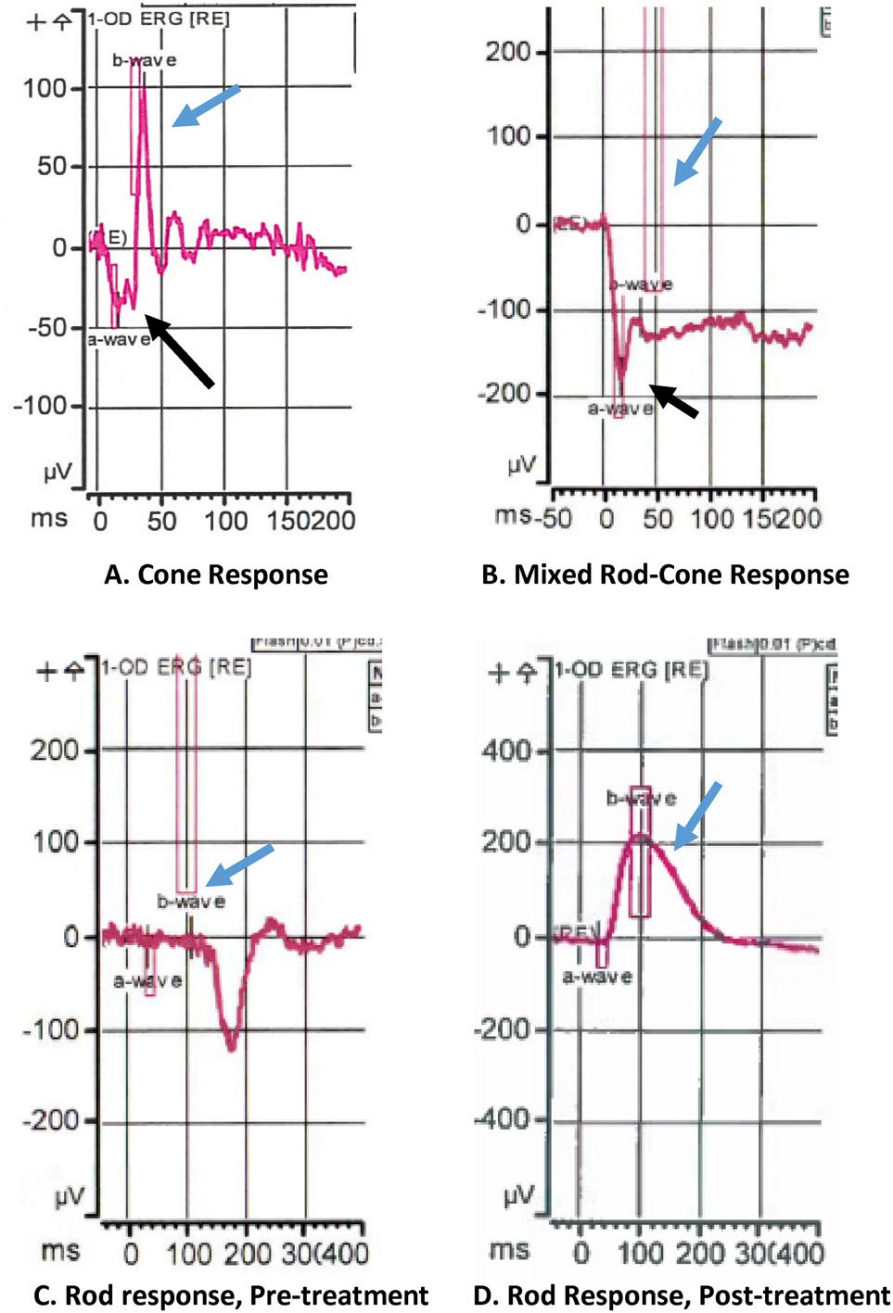
Electroretinogram (ERG) of patient from the case report. ERG measures electrical activity produced by the photoreceptor cells of the retina in response to light stimulus. In melanoma-associated retinopathy (MAR), early in the disease, the response of cone photoreceptors to light is normal with a negative a-wave (black arrow) and a positive *b-wave* (blue arrow) as seen in (**A**), but characteristically, when rod photoreceptor response is tested, it shows abnormal b-wave (blue arrow) which points to bipolar cell dysfunction diagnostic of MAR (**B**). In (**C**) ERG demonstrating severe widespread rod dysfunction in this patient at the time of presentation of symptoms of MAR (absence of b-wave marked by blue arrow) versus (**D**), which shows normalization of the rod bipolar cell dysfunction as shown by the recovery of the b-wave (blue arrow) in response to treatment with PD-1 inhibitors

**Fig. 2:**
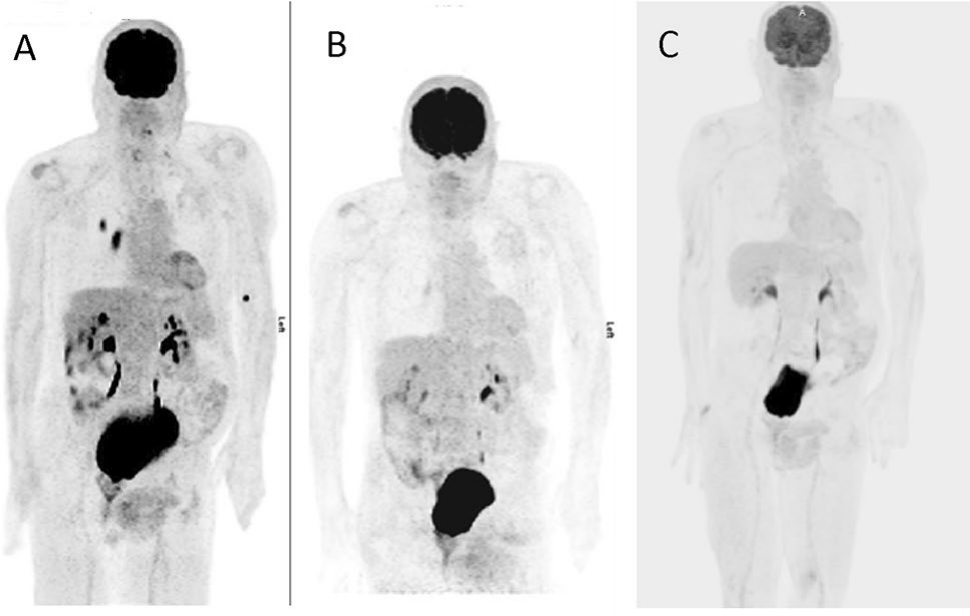
Positron emission tomography which demonstrates. (**A**) hypermetabolic round soft tissue mass within the superior segment of the right lower lobe and hypermetabolic right hilar lymphadenopathy; (**B**) resolution of the hypermetabolic mass in the lung after 6 weeks of initiating pembrolizumab; (**C**) maintained response without development of new hypermetabolic foci two years after treatment with pembrolizumab

The patient’s previous history of melanoma coupled with his symptoms of nyctalopia and shimmering lights raised a concern for the possibility of melanoma-associated retinopathy (MAR). We ordered an electroretinogram (ERG) which revealed prominent reduction of b-waves, indicative of bipolar cell dysfunction—a hallmark of MAR (Fig. 1). The patient was tested negative for retinal anti-recoverin autoantibodies (Athena Diagnostics), neuro-specific enolase and aldolase (Quest Diagnostics). Since onset of MAR often indicates presence of metastatic melanoma, a positron emission tomography–computed tomography (PET–CT) scan was obtained and showed a hypermetabolic soft tissue mass in the right lower lobe of the lung along with hilar adenopathy (Fig. 2). The hilar node was biopsied which was consistent with metastatic melanoma (PD-L1 expression 30% by immunohistochemistry, BRAF-wild type and tumor infiltrating lymphocytes of 20%). Brain magnetic resonance imaging (MRI) did not demonstrate intracranial metastatic disease. We started the patient on pembrolizumab (200 mg IV every 3 weeks) with close monitoring, mainly for ophthalmological complications. The patient developed cutaneous hypopigmentation during therapy without ophthalmic issues. In fact, there was a significant improvement in visual symptoms after two cycles of pembrolizumab (6 weeks after initiating anti-cancer treatment) and subsequent PET–CT scan revealed complete metabolic response of the metastatic disease (Fig. 2). Repeat ERG revealed normalization of b-wave indicating resolution of MAR (Fig. 1). Treatment with pembrolizumab was stopped with the two subsequent PET–CT scans continuing to show complete metabolic response, (patient received a total of 17 cycle of pembrolizumab). His most recent follow-up PET–CT scan (30 months after last dose of pembrolizumab) showed no evidence of recurrent malignant disease. On two-and-a-half-year follow-up after stopping pembrolizumab, the patient remains free of all symptoms that led to the diagnosis of MAR and has good stable vision.

## Discussion

Our report describes resolution of MAR symptoms after the use of PD-1 inhibition which was demonstrated by complete resolution of visual symptoms after the second cycle of pembrolizumab and reversal of the pathogenic ERG changes related to MAR. This approach suggests the safety and efficacy of programmed death-1 inhibitors such as pembrolizumab in the treatment of both malignant melanoma and autobody negative paraneoplastic syndrome of MAR. There was a major concern of exacerbating the underlying paraneoplastic syndrome at the time of treatment initiation, but the undetectable retinal autoantibodies against intracellular proteins suggested that the pathophysiology of MAR was likely B-cell driven. The commercially available panel of retinal autoantibodies does not include antibodies against cell membrane. Moreover, we were not able to perform another antibody test directed at transient receptor potential channel protein 1 (TPRM1) as this test was not commercially available.

There is a lack of consensus regarding treatment of MAR, but it has been centered in the past on two different approaches. The first approach revolves around suppressing the immune system to decrease the immune response; however, corticosteroids have been ineffective in most cases [8]. In addition, limited evidence suggests the efficacy of IVIG as a treatment option centered on modulating the immune system [9]. Nevertheless, the interaction between IVIG and ICI has not been studied and one concern of using IVIG in such patients is its ability to expand T-regulatory cells and enhance their function which could lead to decreased anti-tumor immunity [10, 11]. The other treatment approach of MAR is based on cytoreduction of the tumor bed with surgery [12]. The rationale behind this approach is to decrease melanoma-related antigens by reducing the bulk of the tumor leading to autoantibody clearance and decreased production, but this appears to be clinically irrelevant in the case of metastatic melanoma. Of interest, Keltner et al. suggested that the presence of MAR was associated with prolonged survival in 62 melanoma patients [8]. This has been hypothesized to be mediated by antibodies directed at specific epitopes located on melanoma cancer cell which leads to effective antitumor immunity [13].

The treatment paradigm of advanced non-metastatic and metastatic melanoma has shifted in the last decade from chemotherapeutic agents to targeted therapy and ICI. The 5-year survival data have demonstrated significant improvement of overall survival reaching 52% and 41% with the use of PD-1/CTLA-4 inhibitor combination (nivolumab and ipilimumab) and PD-1 inhibitor pembrolizumab, respectively [14, 15]. However, the use of ICI can be associated with a substantial risk due to over activation of the immune system, which can lead to immune-related adverse events that are usually treated with immunosuppressant including glucocorticoids. Of interest, there is an emerging evidence, although controversial, on the negative impact of glucocorticoids on anti-tumor response when used prior to or in conjunction with ICI.

### The role of early use of glucocorticoids on the efficacy of immune checkpoint inhibitors

There have been no prospective studies to support the role of immunosuppressants such as glucocorticoids on the efficacy of ICI, and most of the evidence is derived from retrospective analysis. For example, in a retrospective study by Fucà et al. the early use of steroids prior to the initiation of ICI in patients with lung cancer was associated with worse outcomes including worse progression-free survival and overall survival [5]. Similarly, in a study by Arbour et al. in 640 patients with metastatic lung cancer who were treated with a single PD-1 inhibitor, there were worse outcomes associated with decreased progression-free survival and overall survival in patients who had received glucocorticoids prior to starting ICI and the authors recommended prudent use of glucocorticoids at the time of initiation of ICI [6]. In the case of metastatic melanoma, controversy exists on the effect of steroids on survival in patients treated with ICI [16, 17]. For example, Faje et al. found that patients with metastatic melanoma who were treated with ICI (ipilimumab) and received high doses of glucocorticoids had worse outcomes including decreased overall survival and time to treatment failure compared to patients who did not receive high doses glucocorticoids [16]. A recent meta-analysis demonstrated a negative impact of the use of corticosteroids on progression-free survival and overall survival in multiple tumor types (including melanoma) in patients who were treated with ICI [18]. As such, patients with MAR could have a compromised anti-tumor response to ICI if they receive glucocorticoids for the treatment of their paraneoplastic syndrome (MAR) prior to the initiation of ICI such as in our case [19]. Therefore, glucocorticoids should be administered with caution in patients receiving ICI until further prospective data are available. This concern has resulted in the exclusion of cancer patients who are on a high dose of glucocorticoids from ICI clinical trials. Given this concern, we attempted to use a new approach by avoiding the use of immunosuppression. We treated our patient with pembrolizumab without the administration of systemic glucocorticoids, which led to a complete and durable resolution of metastatic melanoma and was accompanied by a complete resolution of patient’s visual symptoms related to MAR.

### The role of immune checkpoint inhibitors in aggravating paraneoplastic syndrome

The immune response implicated in the pathogenicity of paraneoplastic neurological syndromes is regarded to be humoral (B-cell mediated) for unknown reasons, and only a minority of these syndromes could be mediated by cellular immunity (T-cell response) [20]. The B-cell driven response targets onconeural surface antigens, while the T-cell driven responses are directed at intracellular proteins. As such, ICI are not expected to exacerbate or provoke a paraneoplastic syndrome mediated by B-cell humoral reaction as the main mechanism of action of ICI is through the engagement of effector T-cells against cancer cells with minimal influence on B-cell function.

Our patient did not develop autoimmune uveitis, retinitis, or autoimmune orbital disease, as some patients on PD-1 inhibitors may develop neurological immune-related adverse events. Contrary to our report, Roberts et al. described a patient who was diagnosed with metastatic cutaneous melanoma and developed MAR with unanticipated fundus findings while receiving treatment with pembrolizumab [21]. The pathological findings were manifested by chorioretinal scars with pigment accumulations developed in the retinal periphery in both eyes [21]. This patient tested positive for anti-retinal autoantibodies against the carbonic anhydrase II, aldolase, and enolase that are directed against intracellular proteins which likely led to T-cell activation and worsening underlying MAR [21]. In contrast, Audemard et al. reported successful treatment of MAR with ipilimumab in one patient [22]. Interestingly, there were no detectable autoantibodies in the previous case (personal communication with the author). The discrepancy of ophthalmologic outcomes of MAR with the use of ICI in different reports may suggest that ICI can play a different role in either exacerbating or resolving symptoms of MAR based on the type of tumoral antigen proteins (intracellular or membranous) and the elicited autoimmune response whether its T-cell or B-cell driven. As such, our patient demonstrated normalization of ERG and resolution of visual symptoms of MAR after initiating ICI. This suggests that cell surface or synaptic antigen-driven antibody response was interrupted by the use of ICI due to priming and proliferation of T-cells leading to tumor volume reduction and subsequently negating reactive B-cell response. Thus far, over 30 different antigens-driven antibodies in the retina have been identified in association with vision loss with most anti-retinal autoantibodies targeting intracellular proteins, and only a few that are directed against membrane proteins [23]. Given the lack of comprehensive testing for anti-retinal autoantibodies and the broad diversity of the different antigens, it becomes more challenging to detect the specific protein with commercially available tests and this can lead to uncertainty for the treating physician about projected ICI treatment outcomes.

Therefore, further research into delineating the heterogeneity of the immunological dysfunction related to MAR is essential, as current standard of care is based on immunotherapy (ICI). It appears that the safety of ICI use in MAR depends on the immunological cellular pathophysiology (B-cell mediated vs T-cell mediated). Our observation along with previously reported cases demonstrate the discrepancy in outcomes in patients with MAR who receive ICI, which suggests the immunological heterogeneity leading to MAR and advocates for further research to understand the immunological response in this paraneoplastic syndrome. The development of clinically available antibody panels directed at intracellular and extracellular retinal antigens could aid in the decision making on treatment approach of metastatic melanoma patients who are diagnosed with MAR and are expected to receive immunotherapy. The rare incidence of MAR and the presence of retinal antibodies in healthy individual controls pose a challenge in establishing a unified approach [24]. Limitations to our report include inability to perform a comprehensive retinal antibody testing and lack of mechanistic insight on the immunological response (i.e., B-cell vs T-cell mediated) in our patient.

In conclusion, this is the first case, to our knowledge, to describe the resolution of autoantibody negative MAR after treatment with programmed death-1 (PD-1) inhibitor in a patient with metastatic melanoma. The possible negative impact of glucocorticoid (used to treat MAR) on ICI anti-tumor response and the potential role of ICI on the resolution of sub-types of paraneoplastic syndrome highlight the importance of multidisciplinary approach when treatment is planned. We believe patients with undetectable retinal-intracellular protein autoantibodies can be safely treated with ICI. The limitations of this study include a single patient and lack of mechanistic insight into the immunological response of MAR in this patient, which warrants further research to support our observation.

## Abbreviations

CTLA-4: Cytotoxic T-lymphocyte-associated protein-4
ERG: Electroretinogram
IVIG: Intravenous immunoglobulins
MAR: Melanoma-associated retinopathy
PD-1: Programmed death-1
PET–CT: Positron emission tomography–computed tomography
